# Supplementary material for: Soil properties and microbial communities of spring maize filed in response to tillage with straw incorporation and nitrogen fertilization in northeast China
Source: PeerJ. 2022 May 13;10:e13462. doi: 10.7717/peerj.13462 (PMC9109688; doi:10.7717/peerj.13462)
Supplement: Supplemental Information 1 — Rotary tillage with straw incorporation (RTS), Plow tillage with straw incorporation (PTS), 0 (CK), 187 (MN) and 337 (HN) kg N ha–1 applied. The values are mean ± standard deviation (n = 3). The groups accounting for 1% are shown, whereas those accounting for <1% are combined (Others). [file peerj-10-13462-s001.docx]

| Properties | RTS | | |  | PTS | | |
| --- | --- | --- | --- | --- | --- | --- | --- |
|  | CK | MN | HN |  | CK | MN | HN |
| Gammaproteobacteria | 17.84±1.07bc | 16.85±0.70c | 20.34±1.14a |  | 18.13±1.62bc | 19.14±1.54ab | 17.68±0.52bc |
| Alphaproteobacteria | 18.31±1.95a | 17.39±0.63ab | 15.93±1.42b |  | 16.16±0.27b | 17.58±0.11ab | 15.73±1.04b |
| Actinobacteria | 13.35±1.52a | 15.03±1.45a | 15.53±3.01a |  | 12.40±0.94a | 13.93±1.91a | 15.45±0.08a |
| Acidobacteriia | 10.19±0.24b | 11.13±0.50a | 9.17±0.28c |  | 7.74±0.59d | 8.94±0.22c | 9.04±0.48c |
| unclassified_Bacteria | 8.60±0.44b | 8.81±0.15b | 8.17±0.40b |  | 9.84±0.19a | 8.46±0.54b | 8.54±0.33b |
| unclassified_Actinobacteria | 6.33±0.62b | 6.69±0.29b | 6.07±1.01b |  | 6.57±0.38b | 6.46±1.32b | 8.33±0.30a |
| Bacteroidia | 3.75±0.53b | 3.62±0.25b | 5.41±0.24a |  | 3.73±0.15b | 4.87±0.65a | 5.12±0.25a |
| Thermoleophilia | 3.21±0.39b | 3.17±0.08b | 3.38±0.33b |  | 3.84±0.30ab | 3.45±0.51b | 4.19±0.30a |
| Deltaproteobacteria | 3.58±0.29a | 2.96±0.14a | 2.80±1.08a |  | 3.70±0.19a | 3.49±0.85a | 2.89±0.27a |
| Subgroup 6 | 1.40±0.12b | 1.07±0.07b | 1.02±0.36b |  | 2.17±0.10a | 1.11±0.32b | 1.15±0.08b |
| AD3 | 1.37±0.10a | 1.39±0.11a | 1.48±0.11a |  | 1.10±0.02b | 1.15±0.13b | 1.10±0.04b |
| Saccharimonadia | 1.08±0.12a | 1.71±0.09a | 1.55±0.99a |  | 1.10±0.14a | 1.10±0.93a | 0.92±0.11a |
| Verrucomicrobiae | 0.97±0.28a | 0.94±0.12a | 0.59±0.48a |  | 1.00±0.22a | 1.01±0.36a | 1.15±0.29a |
| Others | 10.02±0.19b | 9.24±0.22b | 8.55±1.42b |  | 12.51±0.13a | 9.33±1.38b | 8.82±0.41b |
