# Supplementary material for: Soil properties and microbial communities of spring maize filed in response to tillage with straw incorporation and nitrogen fertilization in northeast China
Source: PeerJ. 2022 May 13;10:e13462. doi: 10.7717/peerj.13462 (PMC9109688; doi:10.7717/peerj.13462)
Supplement: Supplemental Information 2 — Rotary tillage with straw incorporation (RTS), Plow tillage with straw incorporation (PTS), 0 (CK), 187 (MN) and 337 (HN) kg N ha–1 applied. The values are mean ± standard deviation (n = 3). The groups accounting for 1% are shown, whereas those accounting for <1% are combined (Others). [file peerj-10-13462-s002.docx]

| Properties | RTS | | |  | PTS | | |
| --- | --- | --- | --- | --- | --- | --- | --- |
|  | CK | MN | HN |  | CK | MN | HN |
| Tremellomycetes | 31.58±2.81c | 58.28±3.02b | 83.37±0.46a |  | 38.71±3.20c | 52.10±8.61b | 58.19±3.82b |
| Sordariomycetes | 27.73±2.59a | 13.85±0.90c | 5.76±0.54d |  | 21.06±4.04b | 14.56±3.06c | 13.57±4.12c |
| Agaricomycetes | 12.04±4.13a | 6.20±1.75ab | 0.67±0.20b |  | 8.04±5.26ab | 12.31±8.46a | 6.49±1.94ab |
| Dothideomycetes | 6.62±1.50b | 6.93±0.41ab | 3.68±0.60c |  | 5.81±1.57bc | 5.73±0.60bc | 9.04±2.10a |
| Leotiomycetes | 3.63±0.60b | 3.97±0.59b | 3.40±0.51b |  | 10.67±2.65a | 4.85±0.86b | 5.11±0.11b |
| unclassified_Fungi | 5.13±0.85a | 2.64±0.75cd | 0.83±0.26e |  | 3.88±0.76b | 3.79±0.62bc | 1.61±0.47de |
| Eurotiomycetes | 5.16±0.57a | 2.84±0.50c | 0.59±0.03e |  | 4.29±0.37b | 2.72±0.75c | 1.80±0.27d |
| Mortierellomycetes | 1.60±0.16bc | 2.14±0.19b | 0.40±0.10d |  | 3.70±0.92a | 2.00±0.16bc | 1.29±0.44c |
| Others | 6.50±2.12a | 3.14±1.11b | 1.30±0.20b |  | 3.83±0.60b | 1.95±0.33b | 2.91±0.71b |
