# Supplementary material for: Soil properties and microbial communities of spring maize filed in response to tillage with straw incorporation and nitrogen fertilization in northeast China
Source: PeerJ. 2022 May 13;10:e13462. doi: 10.7717/peerj.13462 (PMC9109688; doi:10.7717/peerj.13462)
Supplement: Supplemental Information 3 — Rotary tillage with straw incorporation (RTS), Plow tillage with straw incorporation (PTS), 0 (CK), 187 (MN) and 337 (HN) kg N ha–1 applied. The values are mean ± standard deviation (n = 3). The groups accounting for 1% are shown, whereas those accounting for <1% are combined (Others). [file peerj-10-13462-s003.docx]

| Properties | RTS | | |  | PTS | | |
| --- | --- | --- | --- | --- | --- | --- | --- |
|  | CK | MN | HN |  | CK | MN | HN |
| Betaproteobacteriales | 10.93±0.55c | 10.29±0.14bc | 12.87±1.36a |  | 12.70±1.68a | 13.14±0.32a | 12.50±0.54ab |
| unclassified_Actinobacteria | 16.00±1.71bc | 17.89±0.89ab | 16.26±1.11bc |  | 15.31±1.10c | 15.82±0.49c | 18.92±0.62a |
| unclassified_Bacteria | 8.60±0.44b | 8.81±0.15b | 8.17±0.40b |  | 9.84±0.19a | 8.46±0.54b | 8.54±0.33b |
| unclassified_Alphaproteobacteria | 8.27±1.02a | 8.22±0.40a | 7.30±0.62ab |  | 7.81±0.21ab | 7.99±0.17ab | 7.02±0.36b |
| Acidobacteriales | 7.95±0.17b | 8.91±0.41a | 7.16±0.34c |  | 6.04±0.47d | 7.05±0.23c | 7.37±0.41bc |
| Rhizobiales | 5.48±0.54a | 4.86±0.37a | 4.52±0.89a |  | 4.89±0.22a | 5.31±0.70a | 4.87±0.41a |
| Gemmatimonadales | 4.12±0.03b | 4.08±0.12b | 3.73±0.69b |  | 5.83±0.38a | 4.24±0.70b | 4.01±0.42b |
| Gammaproteobacteria | 2.98±0.10a | 3.23±0.26a | 3.80±0.97a |  | 3.45±0.22a | 3.37±0.89a | 2.82±0.13a |
| Myxococcales | 3.21±0.25a | 2.63±0.15a | 2.40±0.69a |  | 3.15±0.21a | 3.08±0.77a | 2.53±0.25a |
| Micrococcales | 1.92±0.13bc | 2.26±0.23bc | 3.57±0.66a |  | 1.55±0.04c | 2.82±0.60ab | 2.94±0.23ab |
| Xanthomonadales | 3.22±0.19a | 2.86±0.44abc | 2.98±0.74ab |  | 1.62±0.11c | 2.07±0.89abc | 1.85±0.11bc |
| Gaiellales | 2.03±0.19b | 1.95±0.06b | 2.00±0.36b |  | 2.36±0.30ab | 2.07±0.35b | 2.77±0.19a |
| Sphingomonadales | 2.14±0.34a | 1.99±0.10ab | 1.82±0.13ab |  | 1.90±0.16ab | 1.84±0.24ab | 1.60±0.21b |
| Chitinophagales | 1.64±0.26b | 1.45±0.14b | 2.25±0.05a |  | 1.59±0.16b | 2.17±0.29a | 2.00±0.08a |
| Sphingobacteriales | 1.49±0.16c | 1.68±0.15c | 2.25±0.26ab |  | 1.41±0.05c | 1.87±0.36bc | 2.31±0.21a |
| Solibacterales | 2.07±0.07a | 2.03±0.07a | 1.90±0.08ab |  | 1.61±0.16c | 1.79±0.01b | 1.58±0.08c |
| unclassified_Subgroup 6 | 1.35±0.11b | 1.03±0.08b | 0.98±0.31b |  | 2.09±0.05a | 1.07±0.29b | 1.13±0.08b |
| Propionibacteriales | 1.16±0.13cd | 1.09±0.12d | 1.27±0.05bc |  | 1.55±0.10a | 1.24±0.07bcd | 1.43±0.10ab |
| Solirubrobacterales | 1.03±0.16b | 1.09±0.04b | 1.20±0.09ab |  | 1.32±0.06a | 1.20±0.13ab | 1.23±0.08ab |
| unclassified_AD3 | 1.34±0.11ab | 1.38±0.13ab | 1.46±0.11a |  | 1.09±0.03c | 1.14±0.14bc | 1.08±0.05c |
| Saccharimonadales | 1.08±0.12a | 1.71±0.09a | 1.55±0.19a |  | 1.10±0.14a | 1.10±0.23a | 0.92±0.11a |
| Others | 12.01±0.73a | 10.55±0.30b | 10.55±1.06b |  | 11.79±0.63ab | 11.17±0.80ab | 10.61±0.61b |
