# Supplementary material for: Soil properties and microbial communities of spring maize filed in response to tillage with straw incorporation and nitrogen fertilization in northeast China
Source: PeerJ. 2022 May 13;10:e13462. doi: 10.7717/peerj.13462 (PMC9109688; doi:10.7717/peerj.13462)
Supplement: Supplemental Information 4 — Rotary tillage with straw incorporation (RTS), Plow tillage with straw incorporation (PTS), 0 (CK), 187 (MN) and 337 (HN) kg N ha–1 applied. The values are mean ± standard deviation (n = 3). The groups accounting for 1% are shown, whereas those accounting for <1% are combined (Others). [file peerj-10-13462-s004.docx]

| Properties | RTS | | |  | PTS | | |
| --- | --- | --- | --- | --- | --- | --- | --- |
|  | CK | MN | HN |  | CK | MN | HN |
| Cystofilobasidiales | 26.75±2.04c | 50.43±3.85b | 79.30±0.52a |  | 32.26±3.59c | 46.65±4.21b | 52.16±4.12b |
| Sordariales | 8.19±1.51ab | 7.63±0.87bc | 3.80±0.55e |  | 9.94±0.88a | 5.92±0.95cd | 5.03±0.49de |
| unclassified_Sordariomycetes | 14.87±1.90a | 3.14±0.50bc | 0.92±0.15c |  | 5.17±1.25b | 4.24±2.06b | 5.17±1.91b |
| Pleosporales | 4.97±1.20ab | 4.60±0.48ab | 2.43±0.52c |  | 4.79±0.97ab | 3.72±0.55bc | 6.48±0.78a |
| Filobasidiales | 4.04±0.83c | 5.83±0.38a | 2.04±0.25d |  | 5.11±0.33ab | 4.55±0.33bc | 5.14±0.67ab |
| Thelebolales | 1.40±0.27d | 2.14±0.18cd | 1.90±0.19d |  | 9.01±1.11a | 3.54±0.42bc | 4.13±0.16b |
| Trechisporales | 4.01±1.62ab | 4.39±10.5ab | 0.33±0.16b |  | 6.59±2.21a | 1.57±0.30b | 1.55±0.47b |
| unclassified_Fungi | 5.13±0.85a | 2.64±0.75cd | 0.83±0.26e |  | 3.88±0.76b | 3.79±0.62ab | 1.61±0.47de |
| Hypocreales | 2.25±0.09a | 2.05±0.30a | 0.57±0.10b |  | 2.87±0.60a | 2.82±0.47a | 2.29±0.63a |
| Chaetothyriales | 3.21±0.25a | 1.98±0.43cd | 0.47±0.04e |  | 2.81±0.44ab | 2.30±0.61bc | 1.59±0.29d |
| Mortierellales | 1.60±0.16bc | 2.14±0.19b | 0.40±0.10d |  | 3.70±0.92a | 2.00±0.16bc | 1.29±0.44c |
| Capnodiales | 1.62±0.35bc | 2.30±0.23a | 1.24±0.11c |  | 1.01±0.42c | 1.99±0.03ab | 2.53±0.62a |
| Agaricales | 0.87±0.26b | 0.06±0.01b | 0.02±0.01b |  | 0.25±0.16b | 8.29±4.12a | 0.42±0.12b |
| Boletales | 2.97±0.87ab | 1.34±0.50bc | 0.01±0.00c |  | 0.06±0.01c | 1.58±0.27bc | 4.15±0.86a |
| Helotiales | 2.21±0.41a | 1.83±0.50ab | 1.49±0.56ab |  | 1.66±0.75ab | 1.26±0.65ab | 0.97±0.27b |
| Eurotiales | 1.95±0.40a | 0.86±0.11c | 0.12±0.05d |  | 1.49±0.16b | 0.42±0.15d | 0.21±0.09d |
| Tremellales | 0.52±0.10b | 1.29±0.28a | 1.35±0.22a |  | 0.60±0.08b | 0.51±0.06b | 0.65±0.19b |
| unclassified_Ascomycota | 1.48±0.62a | 0.98±0.19a | 0.33±0.11a |  | 0.39±0.20a | 0.33±0.15a | 0.69±0.43a |
| Cantharellales | 1.64±0.62a | 0.19±0.06c | 0.25±0.07c |  | 0.71±0.13b | 0.56±0.23b | 0.22±0.04c |
| Others | 10.32±1.64a | 4.18±1.20c | 2.19±0.14c |  | 7.71±1.90b | 3.95±1.10c | 3.74±0.54c |
